# Exchange of polar lipids from adults to neonates in *Daphnia magna*: Perturbations in sphingomyelin allocation by dietary lipids and environmental toxicants

**DOI:** 10.1371/journal.pone.0178131

**Published:** 2017-05-24

**Authors:** Namrata Sengupta, Delaney C. Reardon, Patrick D. Gerard, William S. Baldwin

**Affiliations:** 1 Environmental Toxicology Program, Clemson University, Clemson, SC, United States of America; 2 Biological Sciences, Clemson University, Clemson, SC, United States of America; 3 Mathematical Sciences, Clemson University, Clemson, SC, United States of America; Louisiana State University Health Sciences Center, UNITED STATES

## Abstract

Because xenosensing nuclear receptors are also lipid sensors that regulate lipid allocation, we hypothesized that toxicant-induced modulation of HR96 activity would alter lipid profiles and the balance between adult survival and neonate production following exposure in *Daphnia magna*. Adult daphnids were exposed to unsaturated fatty acid- and toxicant- activators or inhibitors of HR96 and later starved to test whether chemical exposure altered allocation toward survival or reproduction. The HR96 activators, linoleic acid and atrazine, decreased reproduction as expected with concomitant changes in the expression of HR96 regulated genes such as magro. The HR96 inhibitors, docosahexaenoic acid (DHA) and triclosan, increased reproduction or neonate starvation survival, respectively. However, pre-exposure to triclosan increased in neonate survival at the expense of reproductive maturation. Lipidomic analysis revealed that sphingomyelins (SM) are predominantly found in neonates and therefore we propose are important in development. DHA and triclosan increased neonatal SM, consistent with HR96’s regulation of Niemann-Pick genes. While DHA altered expression of magro, Niemann-Pick 1b, mannosidase, and other HR96-regulated genes as expected, triclosan primarily perturbed sphingomyelinase and mannosidase expression indicating different but potentially overlapping mechanisms for perturbing SM. Overall, SM appears to be a key lipid in *Daphnia* maturation and further support was provided by carmofur, which inhibits sphingomyelin/ceramide metabolism and in turn severely represses *Daphnia* maturation and initial brood production. In conclusion, toxicants can perturb lipid allocation and in turn impair development and reproduction.

## Introduction

Zooplankton species such as *Daphnia* occupy an important place in the aquatic food web because they transfer energy rich nutrients from primary producers to higher trophic levels. Seasonal variation may affect the quality of nutrient transfer because of changes in food composition. For example, algal succession alters nutrient composition in part because different algae species produce different amounts of polyunsaturated fatty acids (PUFAs) [1, 2]. Some PUFAs are tightly regulated because they are important in energy metabolism, growth and development, and reproduction through membrane-linked cellular processes [3–5].

A diet rich in PUFAs is crucial in the growth and reproduction of *Daphnia* and other invertebrates [6–8]. For example, diets high in bacteria are not sufficient for proper growth and reproduction because of the lack of sterols and PUFAs in the diet [9]. There are several key PUFAs, including linolenic acid, docosahexaenoic acid (DHA), eicosapentaenoic acid (EPA), linoleic acid, and arachidonic acid (AA) in the diet. Of these AA, DHA, and EPA are considered crucial in growth and reproduction [10–13] although some studies while recognizing AA accumulates in ovaries have not associated AA with improved growth and reproduction [9]. Recently a putative AA receptor named HR97g (relative of HR96) was found highly expressed in the ovaries of adult *D*. *magna* [14]. *D*. *magna* concentrates EPA, DHA, and AA in the body [15], ovaries [16], or eggs [17] when the quantity of food is scarce or the quality of food is poor. However, some studies indicate that DHA is either not detected [17] or rapidly converted to EPA and thus only AA and EPA are concentrated in *Daphnia* [12]. Interestingly, EPA and n-3 fatty acids in general are best associated with fecundity in *Daphnia* species [9, 13]. Most research examined the effects of PUFAs on free fatty acid concentrations in daphnids; however, recent research suggests that polar lipids are also affected by diet and specific polar lipid species may provide protection from toxicant insult [13, 18].

Toxicants can perturb the allocation of lipid resources. Acclimating to toxicants can be an energy expensive process [19, 20] that alters individual demands through behavioral, transcriptional, or metabolic changes [13, 20–22]. Recently, xenobiotics termed obesogens or metabolic disruptors have been found to interfere with lipid allocation and cause obesity and related metabolic disorders such as non-alcoholic fatty liver disease and type-2 diabetes [23–25].

Many of these obesogens perturb transcription factor responses, especially the activity of the nuclear receptors (NRs) that alter lipid utilization and allocation. NRs such as the peroxisome proliferator-activated receptors (PPARs) respond to endogenous fatty acids and obesogens that increase the depuration of fatty acids from the blood into white adipose tissue or the liver [26, 27]. Co-activation of PPARs and its heterodimeric partner retinoid X receptor (RXR) increase obesogen activity and stimulate beta-oxidation of fatty acids [28], and in *Daphnia magna* activation of RXR is shown to perturb nutrient allocation [29] perhaps through its interactions with the ecdysone receptor (EcR) and meυthyl farnesoate receptor (MfR)[30]. Other NRs involved in lipid allocation include the glucocorticoid receptor [31], farnesoid X-receptor [32], and hepatocyte nuclear factor 4a (HNF4**α**) [33]. Receptors first considered xenobiotic-sensors such as the constitutive androstane receptor (CAR) [34, 35], aryl hydrocarbon receptor (AhR) [36] and pregnane X receptor (PXR) [37] also regulate energy balance directly or in part through AMP-kinase [38]. PXR and AhR activation is associated with obesity or fatty liver disease [39, 40], while CAR activation decreases fatty liver disease in mammals [35].

Most of the metabolic disrupting effects of anthropogenic compounds have been investigated in vertebrates, but not extensively studied in invertebrates probably due to a limited knowledge of invertebrate lipid metabolism pathways and their regulation [29]. HR96 is an ortholog of CAR/PXR/VDR [41, 42] that regulates phase I-III detoxification genes and mediates energy metabolism through homeostasis and transport of triacylglycerols and cholesterol [43, 44]. HR96 is also a key regulator of the Niemann Pick type C gene family involved in cholesterol and fatty acid homeostasis (especially sphingolipids). Niemann Pick disease is a lysosomal storage disorder caused by the improper retention of sphingomyelin, and several Niemann Pick genes are sphingomyelinases or sphingomyelin carriers [45]. The metabolism of sphingomyelins (SM) is important in cell signaling and development is often regulated by its metabolites, ceramides, sphingosine, or sphingosine-1-phosphate [46, 47]. In a recently published study with *Daphnia magna*, we showed by principle component analysis (PCA) that four chemicals that alter HR96 in adolescent daphnids such as linoleic acid (LA) (n-6 fatty acid), triclosan, or atrazine, increased retention of SMs, while another, docosahexaenoic acid (DHA), increased retention of phosphatidylcholines (PC). Interestingly, triclosan repressed reproduction by inducing a senescent state under resource limiting conditions that stunted maturation of adolescents to adults [13]. Therefore, xenobiotic-induced changes in lipid profiles are potentially mediated by changes in HR96 activity and may have sublethal effects on resource allocation, development, maturation, and reproduction.

Our goal is to test whether exposure to environmental chemicals that perturb HR96 activity alters lipid allocation. Most studies have investigated the effects of PUFAs on free fatty acid concentrations and reproduction; this study examines whether polar lipids are potentially key determinants in *D*. *magna* health and reproductive outcomes. We will (1) determine differences in the allocation of polar lipids in adults and neonates, (2) determine whether DHA, LA, atrazine, or triclosan perturb polar lipid profiles, and (3) use starvation assays to determine if HR96 activators perturb resource allocation in *D*. *magna* leading to increased starvation survival in lieu of reproduction in comparison to untreated or HR96 inverse agonist exposed daphnids.

## Materials and methods

### *Daphnia magna* culture

*D*. *magna* were maintained in culture at 21–23^°^C in a 16:8 light:dark cycle in moderately hard water. Daphnids were fed *Raphidocelis subcapitata* (Aquatic Biosystems, Fort Collins, CO USA) supplemented with TetraFin fish food (Masterpet Corp., New South Wales, Australia) as described previously [48, 49].

### Chemicals

Atrazine (98.9%) (Sigma-Aldrich, St. Louis, MO USA), Carmofur (>98%) and GW4869 (>90%) (Cayman Chemical, Ann Arbor, MI USA), and zoledronic acid (>98%)(Enzo Life Sciences, Farmingdale, NY USA) were dissolved in 99.7% DMSO (Fisher Scientific, Fair Lawn, NJ, USA). Linoleic acid (LA)(≥ 99%), docosahexaenoic acid (DHA)(≥ 98%), palmitic acid (PA)(99%) and triclosan (97%) (Sigma-Aldrich) stock solutions were dissolved in absolute ethanol (Sigma-Aldrich Chemical Co., Inc, Milwaukee, WI USA).

### Starvation survival

**Fig 1:**
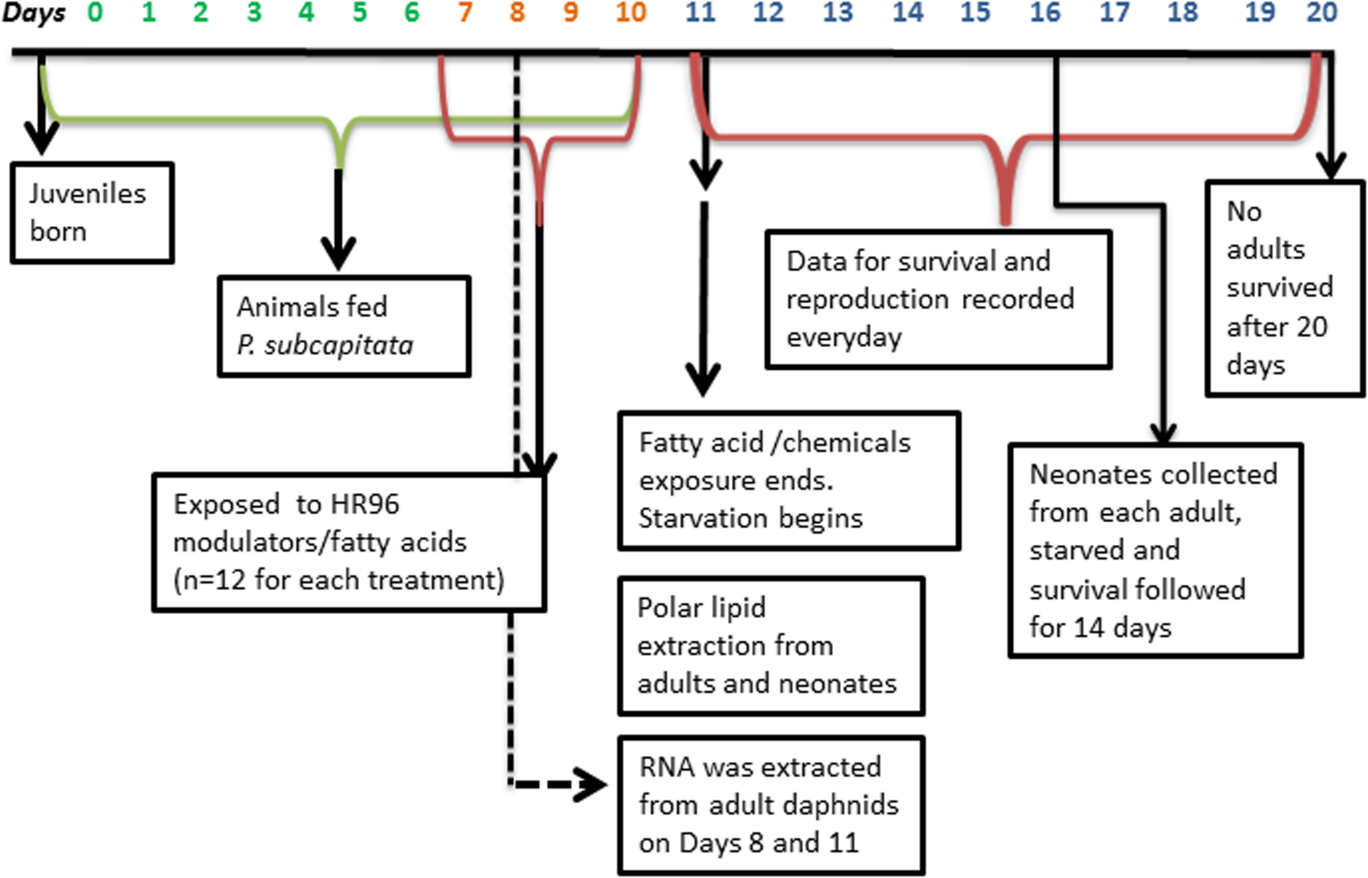
Timeline for chemical exposures, starvation, and polar lipid determinations in *D*. *magna* as described in materials and methods.

*D*. *magna* embryos descend into their brood chamber at around day 7 at 21–23^°^C with the first brood release occurring on day 9–10. Therefore, we exposed 7-day old *D*. *magna* to the fatty acids LA (4 and 8 μM), DHA (2 and 4 μM), and PA (2 and 4 μM), and the xenobiotics atrazine (20 and 40 μM) and triclosan (0.1 and 0.25 μM) (n = 12) for four days as shown in the experimental timeline (**Fig 1)**. A similar experimental design was described previously [13]; however, these studies were performed with juvenile daphnids (day 1) and these studies are performed with adolescent daphnids (day 7). Neonatal and juvenile daphnids (days 1–7) were fed 3 x 10^6^ *R*. *subcapitata*. After day 7, daphnids were fed 3 x 10^6^ *R*. *subcapitata* 2X per day until the starvation phase. Algae was supplemented with 50 μl of an aqueous suspension (2.5 mg/ml) of blended TetraFin fish flakes during the feeding period. Chemical concentrations were chosen based on chronic toxicity tests [13] or atrazine mediated concentration-dependent protection from triclosan and DHA toxicity [50]. All treatment groups and the control group were provided a combination of 0.016% DMSO and 0.004% ethanol as solvent matched controls. On day 11 chemical exposure and feeding was stopped and daphnids were starved to determine if fatty acids or toxicants altered allocation of resources towards survival or reproduction. On day 16 neonates were collected from the adults in each treatment and starvation survival of these neonates was followed for 14 days to determine if fatty acids or toxicant exposure caused a change in allocation of energy resources within the neonates (n = 12). We choose to examine starvation post chemical exposure because we wanted to investigate how exposure changed the allocation of lipids into the ovaries and in turn the oocytes. This process of lipid allocation from the adults to the neonates starts in early stage of egg formation in the ovaries. Once the neonates reach the brood chamber then allocation from the adults to the neonates ends and thus perturbation in lipid allocation in most likely an early process in reproduction. It is estimated to take approximately 1–2 days to produce an oocyte and 2–3 days from oocyte production to neonate release (4–5 days total) [49, 51]. However, on day 15 the number of neonates was insufficient to perform the neonate starvation survival assessment; therefore starvation survival was started on day 16. Starvation survival and reproduction in the adult daphnids was monitored until day 20 as none of the animals survived past day 20. Differences in adult and neonate survival were determined by Fisher’s exact test. Differences in reproduction were determined by one-way ANOVA with a general linear model followed by Fisher’s Least Significant Difference (LSD) (p-value ≤ 0.05) using SAS 9.3 (SAS Institute Inc., Cary, NC).

### Lipid extraction and lipidomic analysis of adult and neonatal daphnids

We exposed 7-day old *D*. *magna* to DHA, LA, atrazine or triclosan for four days (**Fig 1**). Adults and neonates were collected (day 11). Embryos in the brood chamber were separated from the adults to ensure that lipids from the embryos (or soon to be released neonates) were not included in the adult lipid profiles. Polar lipids were extracted from the adult and neonatal daphnids separately with 5 tubes of 3 adult daphnids per treatment group and 30–60 neonates per tube produced from the broods of the 3 extracted adults. Lipid profiles were determined by mass spectrometry (electrospray ionization triple quadrupole mass spectrometer from Applied Biosystems API 4000) analysis at the Kansas Lipidomics Research Center (KLRC) [13, 52]. Changes in lipid profiles were determined by two-way ANOVA followed by Fisher’s LSD as the post-hoc test with GraphPad Prism 6 (GraphPad Software, San Diego, CA). PCA was performed with SAS 9.3 (SAS Institute Inc., Cary, NC) and hierarchical clustering performed with MultiExperiment Viewer (MeV) [53] to confirm associations between specific lipids and chemical treatments.

### RNA extraction and qPCR

Seven-day old daphnids were UT or exposed to DHA, LA, PA, atrazine or triclosan for 24 or 96-hours with 5 or 3 daphnids per beaker, respectively (n = 5 beakers)(**Fig 1; S1 Table** for primers). RNA was extracted with the RNAeasy mini kit (Qiagen, Germantown, MD) and quantified with a spectrophotometer at 260/280 nm. cDNA was synthesized with MMLV reverse transcriptase. qPCR was performed as described previously with *D*. *magna* using 0.25X SYBR Green (Qiagen, Germantown, MD USA) in an iCycler (Bio-Rad, Hercules, CA USA) to quantify expression [11, 54]. Statistical differences in gene expression were determined by one-way ANOVA followed by Fisher’s LSD GraphPad Prism 6 (GraphPad Software) with a p-value ≤ 0.05 (n = 5).

### Acute and chronic toxicity tests

Forty-eight hour acute and 21-day chronic toxicity tests were performed with GW4869, a neutral sphingomyelinase inhibitor [55], zoledronic acid [56], an acid sphingomyelinase inhibitor, and carmofur, an acid ceramidase inhibitor [57]. The control group received DMSO at 0.01%. For acute toxicity, less than 24-hour old daphnids were used with four daphnids per treatment beaker and 5 beakers per exposure group [58]. LC50 values and 95% confidence intervals were determined from sigmoidal dose response curves generated by GraphPad Prism 6 (GraphPad Software). Reproductive toxicity was determined by exposing <24-h *D*. *magna* neonates to these compounds in a 21-day standard chronic toxicity test [59] with renewals every other day. Daphnids were fed 3x10^6^ *R*. *subcapitata* cells supplemented with 50 μl of an aqueous suspension of blended TetraFin fish flakes at 2.5 mg/ml dry weight once a day for the first seven days and twice a day for the remaining fourteen days as described previously [13]. Statistical differences in fecundity were determined by one-way ANOVA with GraphPad Prism 6 (GraphPad Software) with Fisher’s LSD used as the post-hoc test (p ≤ 0.001).

## Results

### Starvation survival and fecundity in adult *D*. *magna*

**Fig 2:**
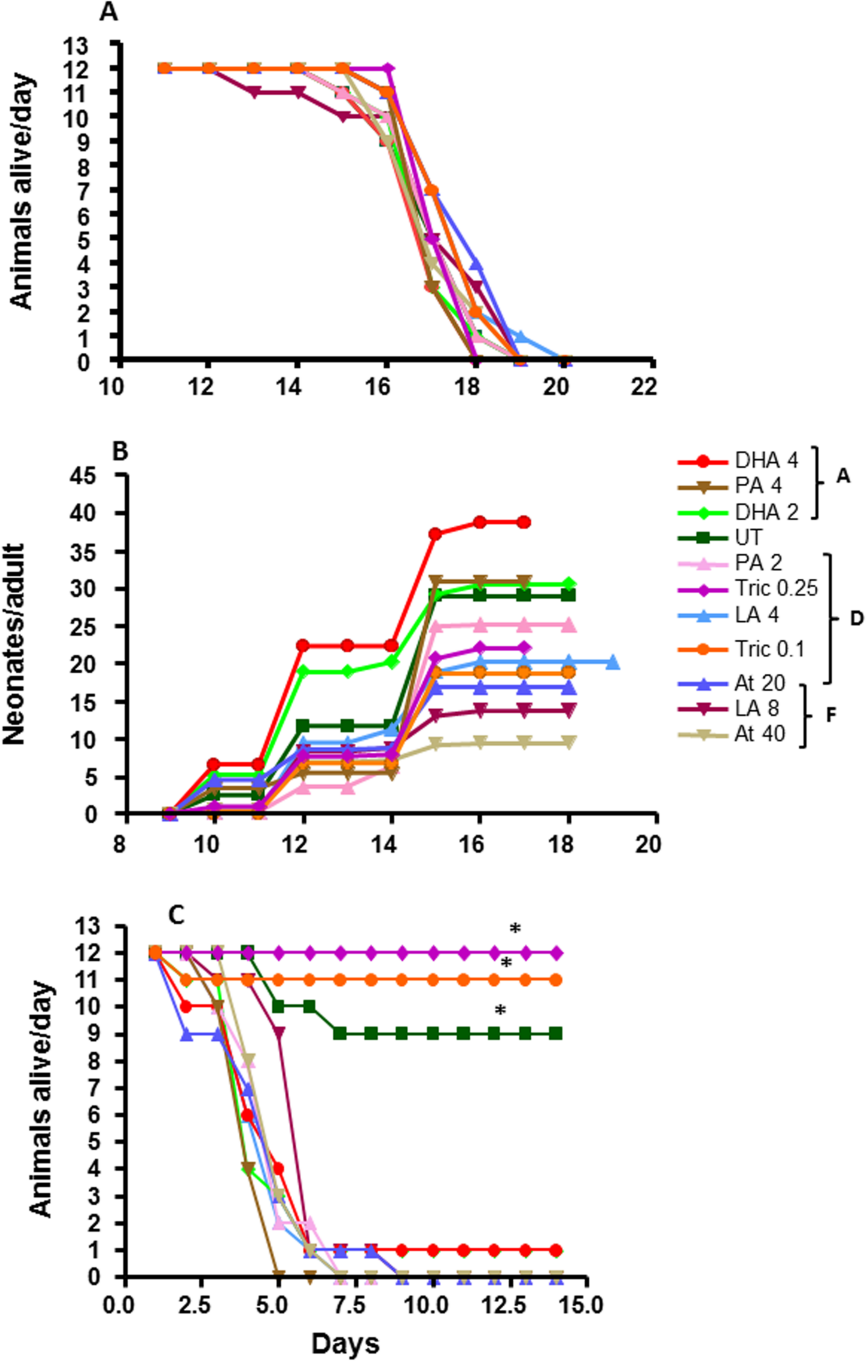
**Adult starvation survival (A), reproduction (B) and neonate starvation survival (C) in daphnids exposed to DHA, LA, PA, atrazine or triclosan.** Data are presented as number of daphnids alive per day (A and C) or number of neonates per adult daphnid (B). Survival of daphnids was analyzed using Fishers 2x2 test (n = 12). An asteriak represents differences from DHA, LA, PA and atrazine groups (p<0.05). Reproduction (B) was analyzed using one-way ANOVA with a general linear model followed by Fisher’s LSD (n = 12) (p<0.05), with higher reproduction observed in group A than UT daphnids, and lower reproduction in groups D and F compared to UT and group A treated daphnids (n = 12).

Previous research demonstrated that dietary lipids and toxicants alter starvation survival and fecundity in juvenile *D*. *magna*. Therefore, we wanted to determine if specific lipids (DHA, LA) are associated with development, reproduction, or survival and examine potential compromises in lipid exchange between adults and their broods under xenobiotic stress. Unexpectedly, pre-exposure of 7-day old *D*. *magna* to different lipids or toxicants did not significantly perturb starvation survival (**Fig 2A**). All of the daphnids in each group died between 18–20 day-old or 7–9 days after starvation was initiated.

However, chemical and lipid exposure perturbed fecundity (**Fig 2B**). In general, atrazine and to a lesser extent triclosan reduced fecundity, while DHA increased fecundity relative to the other groups. Atrazine is the only chemical that reduces reproduction at the concentrations tested under normal, non-starvation conditions [13]. Interestingly, while not statistically significant, higher reproduction was associated with shorter lifespan as the DHA and PA groups that showed the highest fecundity also died youngest (at 18 days-old).

Neonates collected on day 16 from each of the pre-exposed, starved adults were followed for starvation-survival for fourteen days. All of the fatty acid (DHA, LA and PA) and atrazine exposures reduced offspring starvation survival (**Fig 2C**). In contrast, maternal triclosan exposure significantly increased neonate survival time. The daphnids did not develop probably due to the lack of food, but were able to survive in a senescent-like state. Similarly, in our previous study juvenile daphnids exposed to triclosan and then starved at 5-days old did not reproduce or mature to adults but were able to survive in a senescent-like state while other treatment groups continued to develop and reproduce, but with a much shorter lifespan [13]. Thus, the combination of starvation and triclosan is increasing starvation survival but greatly delaying maturity and reproduction by initiating a senescent-like state.

### Fatty acids and toxicants decrease phosphatidylcholine and increase sphingomyelin levels in neonatal daphnids

**Fig 3:**
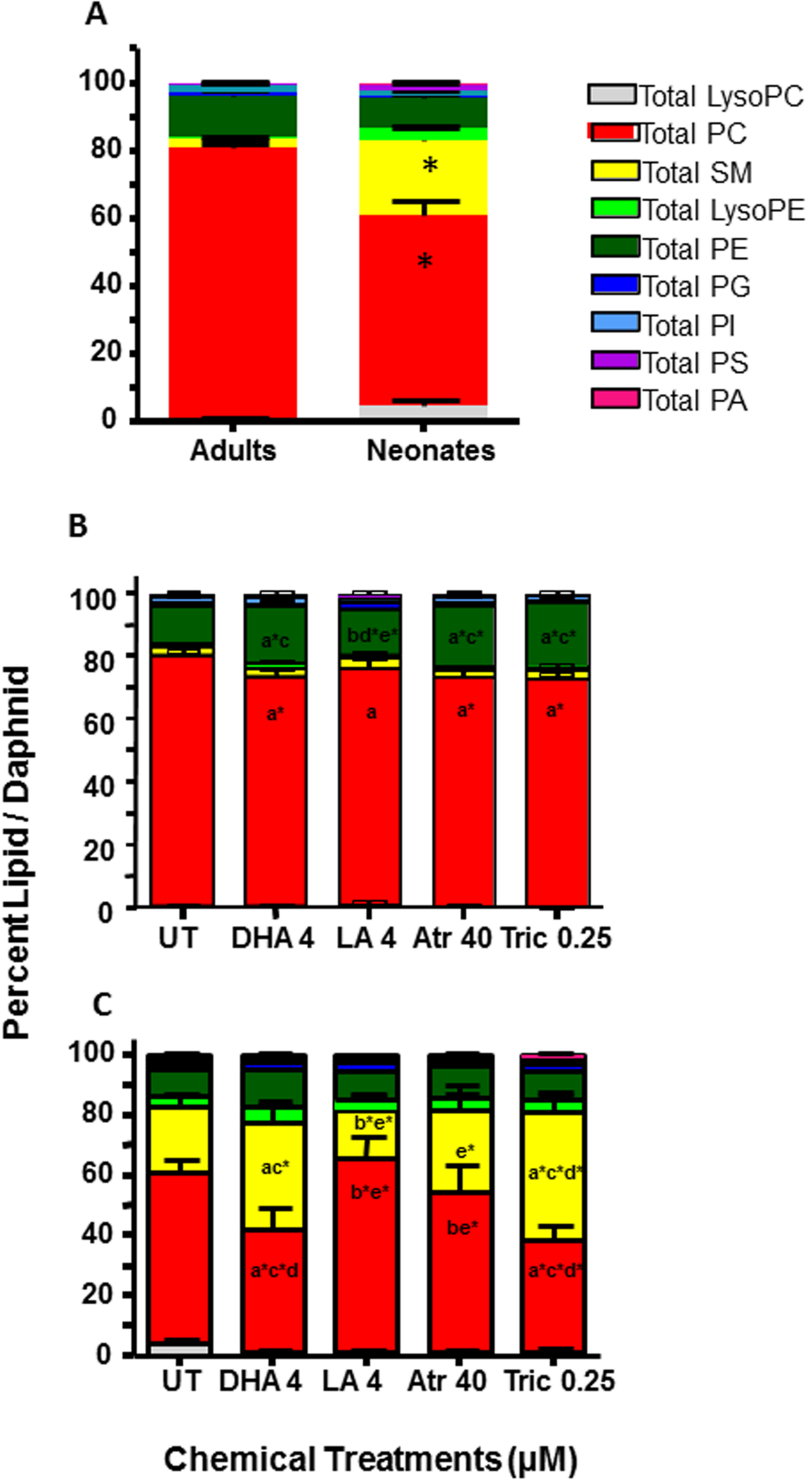
Relative levels of different polar lipid classes in adult and neonatal daphnids. (A) Relative concentrations of different classes of polar lipids in (A) untreated adult and neonatal daphnids, (B) treated adult daphnids, and (C) treated neonatal daphnids. Phosphatidylcholine (PC), phosphatidylserine (PS), phosphatidylinositol (PI), phosphatidylglycerol (PG), phosphatidylethanolamine (PE), phosphatidic acid (PA), sphingomyelin (SM). Data are analyzed by two-way ANOVA followed by Fisher’s LSD as the post-hoc test (n = 5). ‘An a’ is different from UT, ‘b’ from DHA, ‘c’ from LA, ‘d’ from atrazine (Atr), ‘e’ from triclosan (Tric). No asterisk indicates a p<0.01 and an asterisk indicates a p<0.001. All significant data is p < 0.0001 in (A).

Neonatal (1-day old) daphnids preferentially sequester sphingomyelin compared to adult (11-day old) daphnids at the expense of phosphatidylcholine (PC) (**Fig 3A**). For example, in neonates PC formed 56.4% of the lipidome and SM formed 21.8%, an 8.1X increase over adults. In contrast, the adult lipidome (PC 79.8%, SM 2.7%) looks very similar to what was observed previously in adolescents (5-day old daphnids; PC 75%, SM 4.4%)[13]. Therefore, SM levels must drop significantly between days 1 and 5, suggesting a role for the sphingomyelin pathway in instar development.

We were interested in whether there are fatty acids associated with starvation survival or reproduction. Therefore, we hypothesized that dietary lipids and toxicants perturb the exchange of lipid species between reproductive adults and their broods with the potential that some lipid species may be associated with survival and others with reproduction. Of special interest are the PC and SM because their relative concentrations are different in adults than neonates. Triclosan-exposed adult daphnids that showed reduced reproduction, bioconcentrate the most total polar lipid content primarily through increased PC and PE (**Table 1**). Triclosan and atrazine-exposed daphnids also show poor lipid accumulation in their broods (**Table 2**) and this is associated with poor reproduction. Fatty acid treated daphnids (LA, DHA) surprisingly retained the least amount of polar lipids in the adults (**Table 1**); however, some of these lipids are allocated to the neonates as the LA-treated adults produced neonates with the highest levels of total polar lipids and PCs (**Table 2**) even though the LA-treated adults did not reproduce well indicating an increase in lipids/daphnid.

**Table 1:** Quantity of each lipid group found in polar lipids extracted from treated and untreated adult *D*. *magna*.

Lipid	UT £	DHA (4μM)	LA (4μM)	Atr (40 μM)	Tric (0.25 μM)
LysoPC	0.042±0.018	0.031±0.008	0.030±0.044	0.031±0.013	0.031±0.017
PC	4.210±0.427^c^^**^	3.558±1.396^e^^*^	2.853±1.324^a^^**^^d^^*^^e^^**^	3.811±0.694^c^^*^^e^	4.635±1.359^b^^*^^c^^**^^d^
SM	0.145±0.028	0.117±0.028	0.104±0.061	0.120±0.039	0.166±0.043
LysoPE	0.038±0.014	0.065±0.023	0.029±0.012	0.058±0.019	0.065±0.018
PE	0.635±0.155	0.864±0.278	0.567±0.303^e^	1.028±0.249	1.306±0.367^c^
PG	0.070±0.019	0.028±0.022	0.050±0.039	0.030±0.009	0.039±0.014
PI	0.103±0.044	0.109±0.032	0.05±0.031	0.101±0.017	0.087±0.056
PS	0.034±0.010	0.037±0.012	0.029±0.019	0.038±0.007	0.028±0.010
PA	0.008±0.002	0.009±0.001	0.009±0.006	0.011±0.003	0.012±0.002
Lipids	5.286±0.619^c^^**^^d^^*^	4.818±1.715^c^^*^^e^^**^	3.721±1.584^a^^**^^b^^*^^d^^**^^e^^**^	5.227±0.610^c^^**^^e^^*^	6.368±1.816^a^^*^^b^^**^^c^^**^^d^^*^

£ Data presented as mean nmol lipid/daphnid +/- SEM. Statistical differences determined by two-way ANOVA followed by Fisher’s LSD as the post-hoc test,

p<0.05

*p<0.01

**p<0.001 (n = 5).

‘a’ treatment different than UT

‘b’ treatment different than DHA

‘c’ treatment different than LA

‘d’ treatment different than Atr

‘e’ treatment different than Tric

**Table 2:** Quantity of each lipid type found in the polar lipids extracted from treated and untreated neonatal *D*. *magna* (per brood).

Lipid	UT £	DHA (4 μM)	LA (4 μM)	Atr (40 μM)	Tric (0.25 μM)
LysoPC	0.053±0.052	0.012±0.013	0.013±0.008	0.010±0.010	0.018±0.027
PC	0.573±0.370	0.277±0.175^c^^**^	0.791±0.642^b^^**^^d^^*^^e^^*^	0.384±0.437^c^^*^	0.323±0.409^c^^*^
SM	0.230±0.176	0.287±0.256	0.140±0.054	0.127±0.065	0.248±0.113
LysoPE	0.039±0.025	0.043±0.030	0.033±0.009	0.025±0.016	0.027±0.028
PE	0.087±0.051	0.090±0.057	0.090±0.019	0.061±0.043	0.078±0.099
PG	0.008±0.005	0.017±0.027	0.023±0.007	0.003±0.002	0.012±0.006
PI	0.012±0.009	0.005±0.004	0.014±0.010	0.006±0.006	0.011±0.017
PS	0.017±0.014	0.006±0.006	0.010±0.004	0.006±0.004	0.004±0.004
PA	0.006±0.002	0.009±0.006	0.007±0.004	0.007±0.001	0.008±0.004
Lipids	1.025±0.636^d^^*^	0.747±0.465^c^	1.122±0.687^b^^d^^*^^e^^*^	0.629±0.526^a^^*^^c^^*^	0.728±0.684^c^^*^

£ Data presented as mean nmol lipid/brood +/- SEM. Statistical differences determined by two-way ANOVA followed by Fisher’s LSD as the post-hoc test,

p<0.05

*p<0.01

**p<0.001 (n = 5).

‘a’ treatment different than UT

‘b’ treatment different than DHA

‘c’ treatment different than LA

‘d’ treatment different than Atr

‘e’ treatment different than Tric

Relative levels (percent total signal) of each lipid type were also examined given the differences in total lipids between treatments, and between adults and their broods. Different phospholipids are also made from other phospholipids, thus examination of percent can reveal pathway perturbations. Both toxicants and fatty acids significantly lowered PC levels in comparison to the UT adult daphnids (**Fig 3B**). Adult PE levels were significantly lower in UT and LA groups in comparison to the DHA, atrazine and triclosan groups. In contrast, the broods showed increased relative SM levels when exposed to DHA or triclosan, primarily at the expense of PCs (**Fig 3C**). This is an interesting result given that SMs are associated with neonates, DHA and triclosan are HR96 inhibitors, DHA and triclosan alter reproduction (although in opposing directions), and triclosan caused senescence or delayed maturity (**Fig 2**)[13].

**Fig 4:**
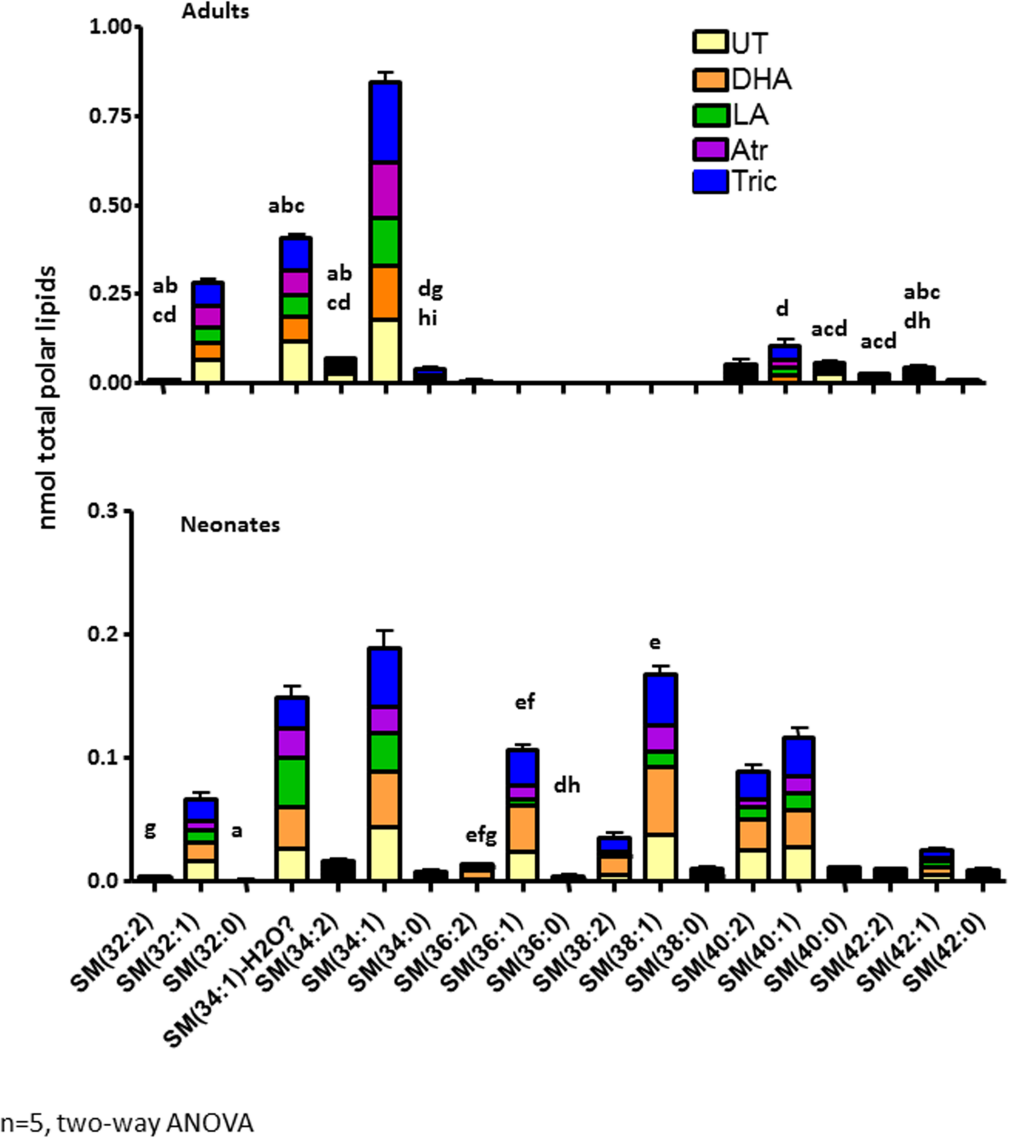
Changes in sphingomyelin (SM) composition among different exposure groups in adult and neonatal daphnids. Changes in concentrations (nmol) of individual SM species following chemical exposure (UT, DHA, LA, atrazine and triclosan) in adult and neonatal daphnids were analyzed by two-way ANOVA followed by Fisher’s LSD (n = 5) (p<0.05). An ‘a’ refers to UT different from DHA, ‘b’ refers to UT different from LA, ‘c’ refers to UT different from Atr, ‘d’ refers to UT different from Tric, ‘e’ refers to DHA different from LA, ‘f’ refers to DHA different from Atr, ‘g’ refers to DHA different from Tric, ‘h’ refers to Tric different from LA, and ‘i’ refers to Tric different from Atr.

Investigation of individual SM species revealed that several mid-weight SMs are neonate specific (i.e. 36:0, 36:1, 38:1 and 38:2)(**Fig 4**). Total polar lipid concentrations among neonates reveal that DHA caused significant increases in SM species 36:1 and 36:2 in comparison to LA and atrazine (p<0.01), and triclosan caused significant increases in 36:0, 36:1 and 36:2 (p<0.05) in comparison to UT, LA, and atrazine exposures (**Fig 4**). Among adults, concentrations of specific SM species such as 32:2, 34:2, 40:0, 42:1 and 42:2 were different in the control group compared to the treatment groups. In summary, several mid-weight SMs are neonate specific and these and other SMs are altered in opposing directions by LA compared to DHA and triclosan.

**Fig 5:**
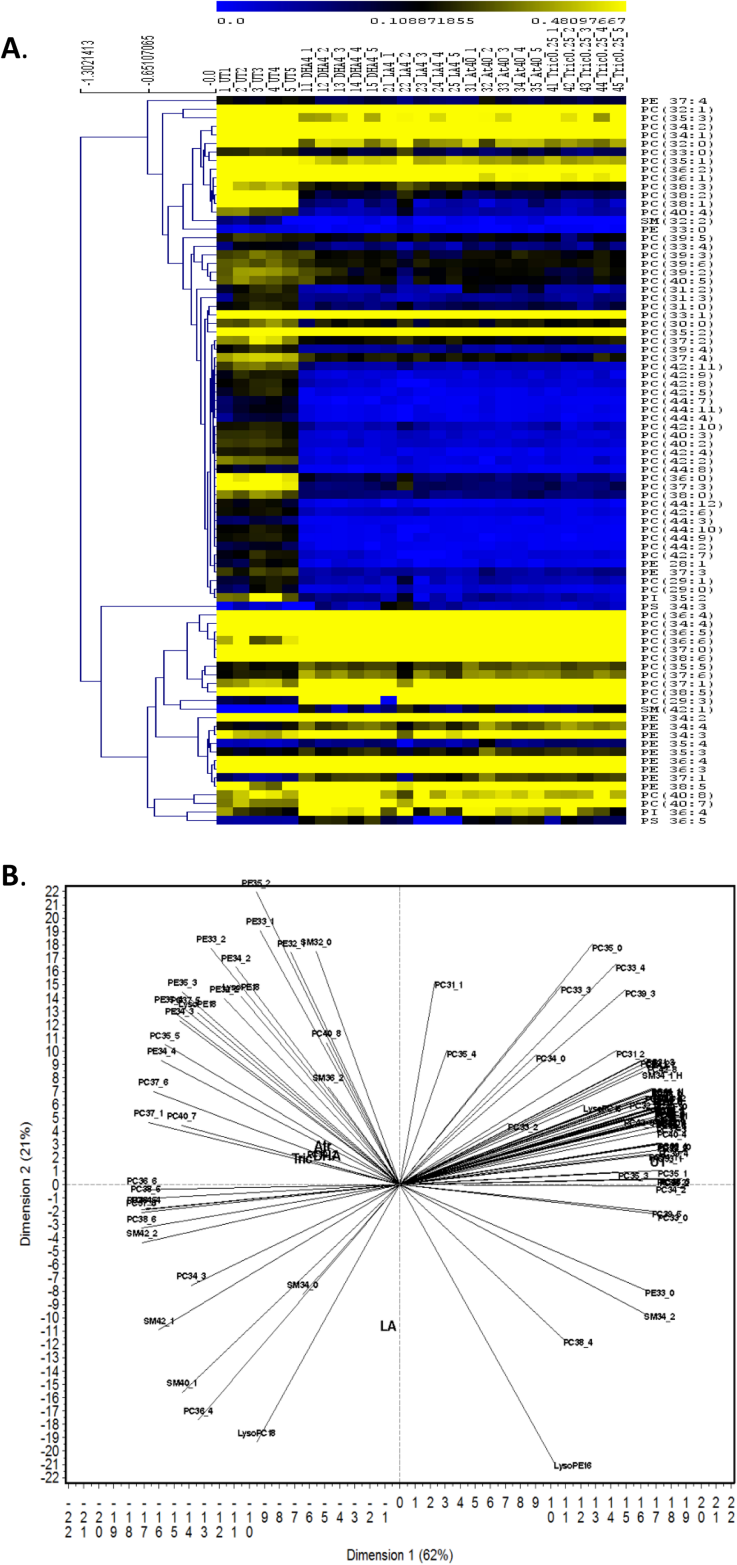
Hierarchal clustering and principle component analysis in adult *Daphnia magna*. **(A)** Hierarchal Clustering (HCL) was used to cluster and visualize significant changes in the percentage signal of individual lipids in the adult daphnids using MultiExperiment Viewer (MeV). One–way ANOVA (p<0.01) with MEV was used to identify significantly altered lipid species. **(B)** Principle component analysis (PCA) demonstrates associations between chemical exposures and lipid profiles in adult *D*. *magna*. Variability among the polar lipids was observed following chemical treatment.

**Fig 6:**
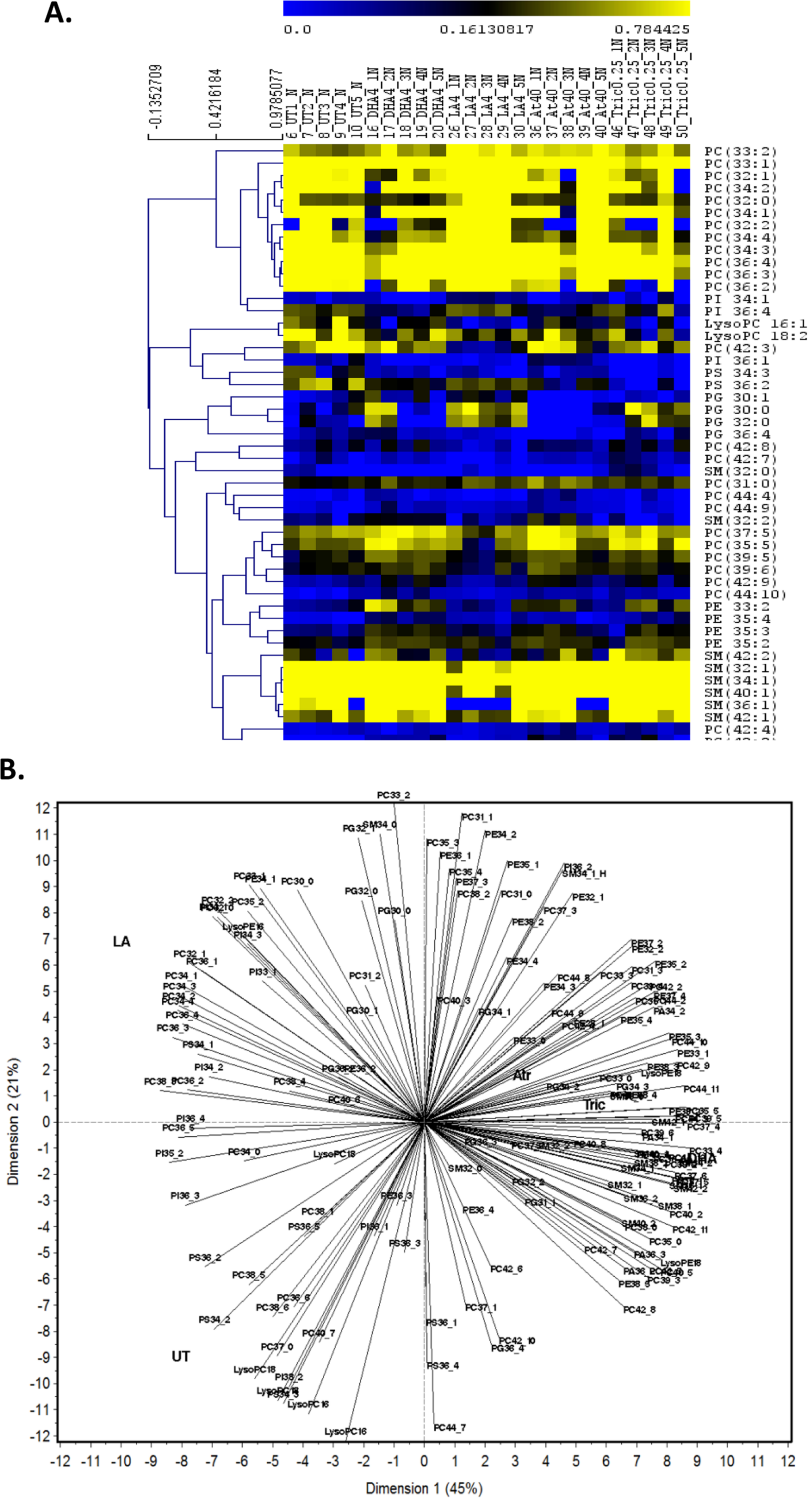
Hierarchal clustering and principle component analysis in neonatal *Daphnia magna*. **(A)** Hierarchal Clustering (HCL) was used to cluster and visualize significant changes in the percentage signal of individual lipids in the adult daphnids using MultiExperiment Viewer (MeV). One–way ANOVA (p<0.01) was used to identify significantly altered lipid species. **(B):** Principle component analysis (PCA) demonstrates associations between chemical exposures and lipid profiles in neonatal *D*. *magna*. Variability among the polar lipids was observed following chemical treatment.

In addition, we investigated all measured polar lipid species (**S2 Table**) following chemical treatment or life stage by hierarchical clustering and Principal Component Analysis (PCA) in adults (**Fig 5**) and neonates (**Fig 6**). Hierarchical Clustering showed that the reduction in PC content in adults is caused by a reduction in many individual PCs following chemical treatments. Hierarchal clustering also revealed that among PE species the ones with 34–38 carbons are increased in adults; whereas PE 37:3, 37:4, 28:1 and 33:0 are decreased in adults. Only two SM species are altered in adults, 32:2, which is produced at very low concentrations, and 42:1, which is increased in several treatment groups. Interestingly, PCA of the adult data sets reveals that DHA, atrazine and triclosan alter adult and neonate lipidomic signatures in a similar fashion, and in an opposing fashion to LA- and UT-exposed adult and neonatal daphnids (**Figs 5 and 6**).

PCA of lipids from neonates confirmed the association of neonates with SM, especially neonate specific SM with triclosan and DHA exposures. SMs with one double bond (32:1, 34:1, 36:1 40:1 and 42:1) are highly expressed in all groups with the exception of SM 36:1 and 42:1 that have low production in the LA group (**Fig 6**). In addition, neonates released from LA exposed daphnids have higher levels of PC and total lipids compared to other groups (**Table 2**) and several low and mid-weight PC species (35:5, 37:5, 39:5 and 39:6) are associated with LA in the PCA bi-plot (**Fig 6**). Therefore, PC’s appear to be associated with lower reproductive outcome potentially because of an increase in n-6 fatty acids that are not as crucial to reproductive health as the n-3 fatty acids [16]. Overall, similar lipid profiles are observed in neonates and adults with perturbations in several phospholipid classes such as PCs, PEs, and SMs.

### Perturbations in the regulation of genes involved in lipid absorption and sphingomyelin metabolism

In addition to detoxication genes, *Drosophila* HR96 regulates the expression of genes involved in cholesterol and triacylglycerol absorption and sphingomyelin metabolism in *Drosophila* [43, 60]. Given the changes measured in sphingomyelin levels as daphnids matured or were treated with DHA or triclosan, we investigated the expression of several genes in this pathway to determine age-dependence and chemical exposure perturbations. Several genes were altered in an age-dependent manner. Of special interest, acid sphingomyelinase 3A significantly increased at 4 days of age, which may explain the loss of sphingomyelins between neonate and adult stages (**Table 3**).

**Table 3:** Age-dependent expression of genes in untreated *D*. *magna*.

Gene	2-day old£	4-day old	7-day old	14-day old
**HR96**	1.00 ± 0.26	1.30 ± 0.17	1.95 ± 0.63	1.11 ± 0.09
**magro**	1.00 ± 0.64	0.65 ± 0.67	0.66 ± 0.67	0.84 ± 0.92
**mannosidase**	1.00 ± 0.69	0.55 ± 0.42	0.52 ± 0.37	6.11 ± 4.34^a^^b^^c^
**NPC1b**	1.00 ± 0.24	1.15 ± 0.45	1.80 ± 0.71^a^	2.52 ± 0.61^a^^b^
**sphingomyelinase**	1.00 ± 0.33	2.75 ± 0.51^a^^c^^d^	1.41 ± 0.41	1.37 ± 0.37
**Cer2**	1.00 ± 0.53	1.39 ± 0.46	1.51 ± 1.01	0.53 ± 0.29^c^

£Data presented as mean relative expression +/- SEM. Statistical differences determined by one-way ANOVA followed by Fisher’s LSD as the post-hoc test (p<0.05) (n = 4–5)

‘a’ expression different than 2-day old

‘b’ expression different than 4-day old

‘c’ expression different than 7-day old

‘d’ expression different than 14-day old

**Fig 7:**
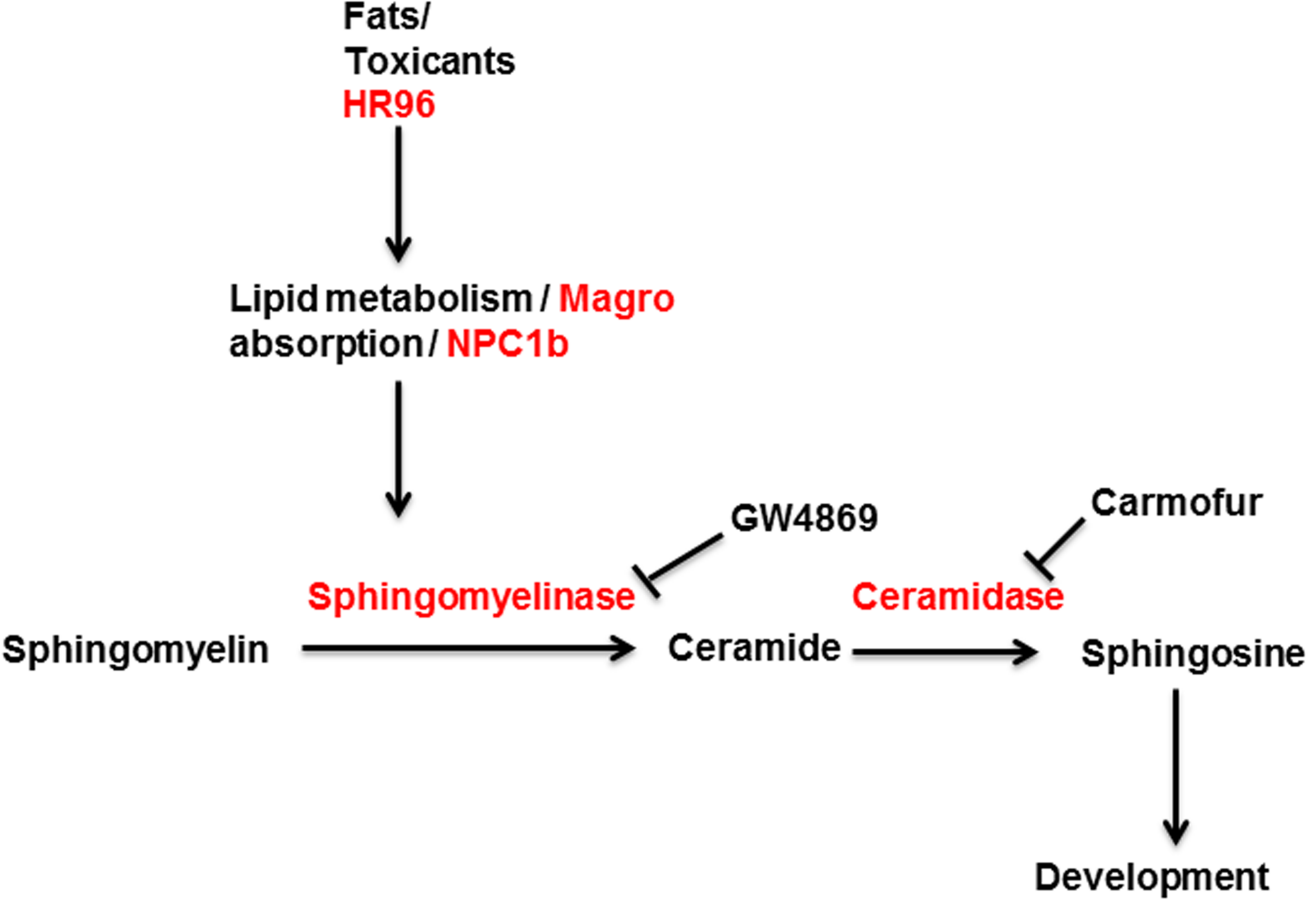
Sphingomyelin metabolism pathway. Genes regulated by HR96 play important roles in breakdown of sphingomyelin to ceramide and sphingosine.

**Fig 8:**
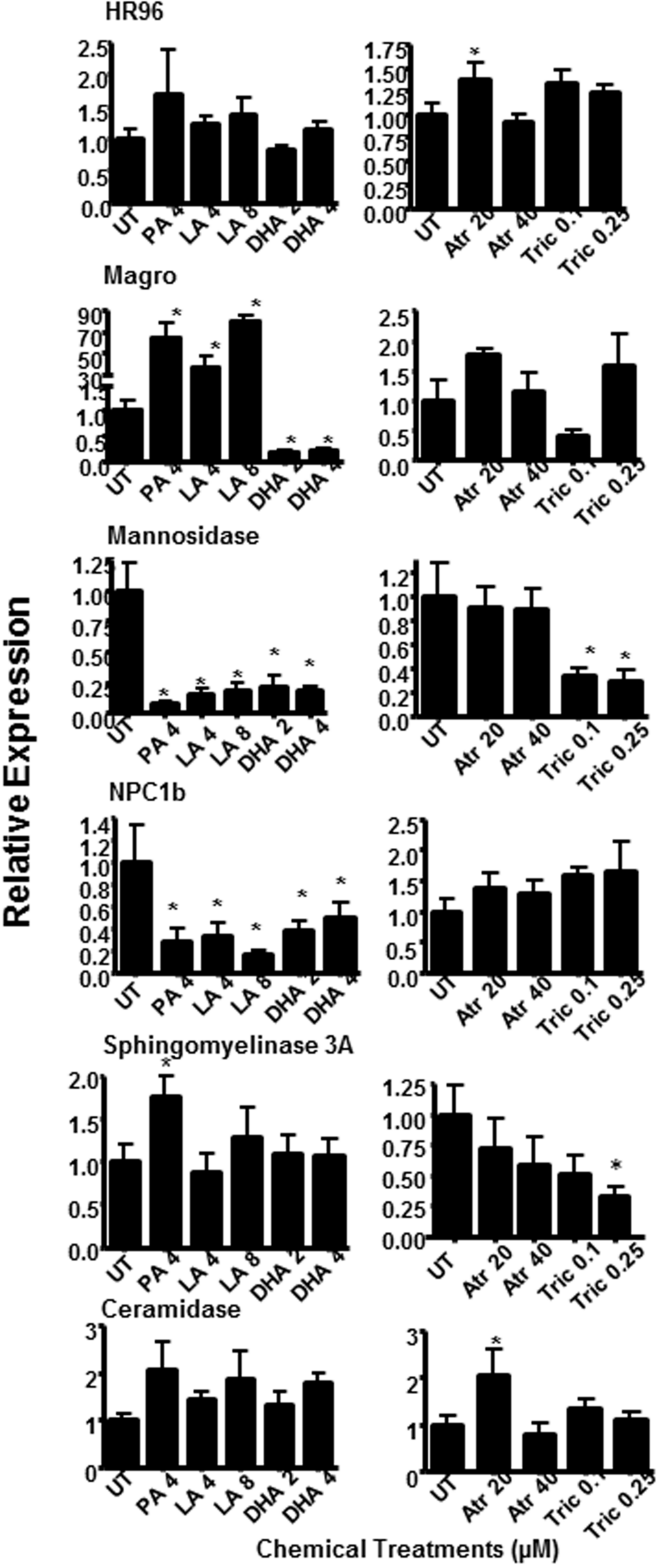
Altered expression of the HR96-regulated genes magro, mannosidase, NPC1b, sphingomyelinase, in addition to ceramidase in 8 day-old *D*. *magna* exposed to fatty acids or toxicants for 24 hours. Statistical significance was determined by one-way ANOVA followed by Fisher’s LSD with *p ≤0.05 considered significant. Data are provided as mean ± SEM (n = 5).

**Fig 9:**
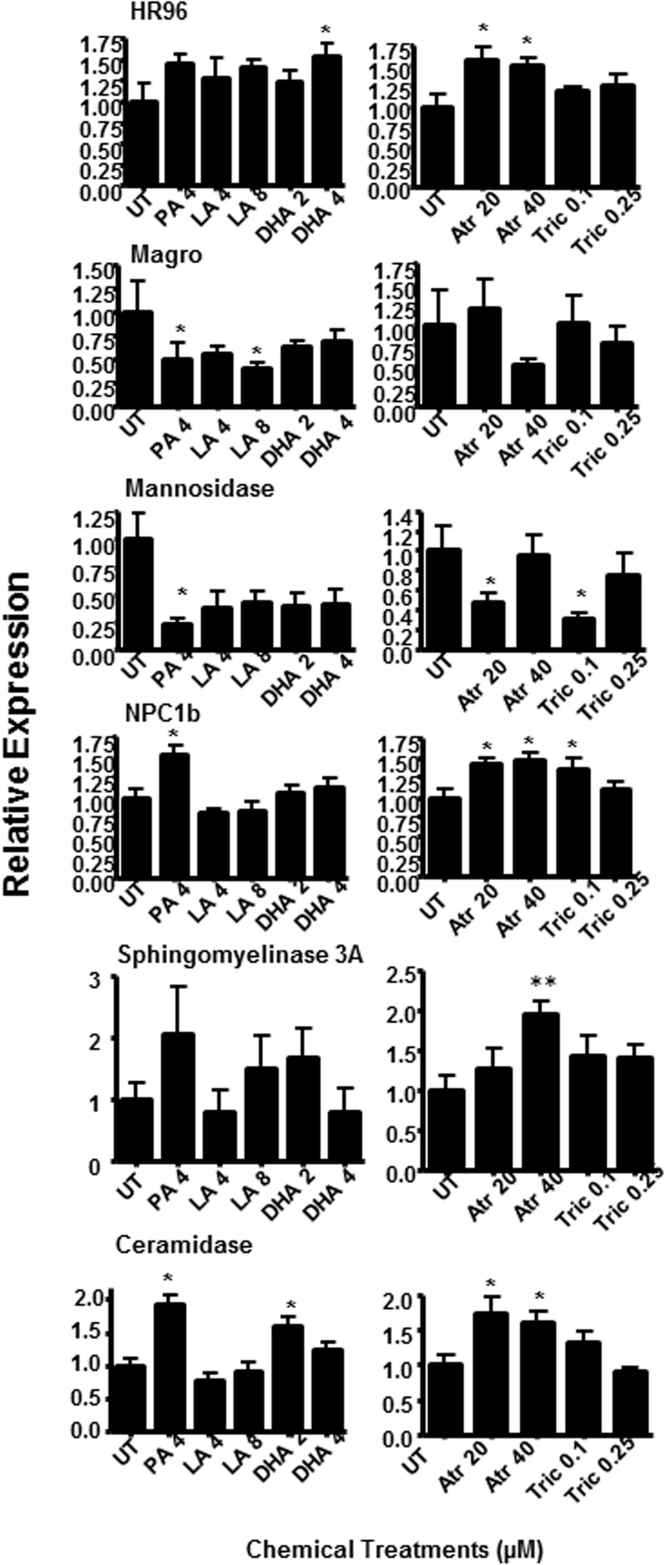
Altered expression of the HR96-regulated genes magro, mannosidase, NPC1b, sphingomyelinase, in addition to ceramidase in 8 day-old *D*. *magna* exposed to fatty acids or toxicants for 96 hours. Statistical significance was determined by one way ANOVA followed by Fisher’s LSD test with *p ≤0.05 considered significant. Data are provided as mean ± SEM (n = 5).

Seven-day old daphnids were treated for either 24 or 96 hours (**Fig 1**). Expression of HR96 and HR96-regulated genes involved in sphingomyelin/sphingosine metabolism (**Fig 7**) were quantified by qPCR [43, 60]. The regulation of ceramidase (Cer2) by HR96 is not known, but we examined its expression because it is important in sphingosine production (**Fig 7**). After 24 hours of exposure, magro, a biomarker of HR96 activation is increased by PA and LA 45-85X, and down-regulated by DHA, an inverse agonist, 5X (**Fig 8**). Lysosomal mannosidase, which is also a biomarker of HR96 and also associated with lysosomal storages diseases [43, 60, 61] was down-regulated by activators as expected but also down-regulated by inverse agonists. NPC1b was down-regulated by all the lipid treatments when we expected induction following exposure to activators. This may indicate similar regulation of these genes by all lipids or differences between *Drosophila* and *Daphnia*. After 96 hours of exposure to the chemicals, the transcriptional effects of the lipids were not as strong (**Fig 9**). However, the toxicants now showed greater effects than after 24 hours of exposure. The potent HR96 activator, atrazine [50] increased HR96, NPC1b, and ceramidase expression and down-regulated mannosidase as originally hypothesized. However, magro was not regulated by atrazine. Sphingomyelinase 3A was induced as expected by atrazine and reduced as expected by triclosan. Overall, many of the genes in the sphingomyelin metabolism pathway were perturbed by these HR96 activators and inhibitors.

### The ceramidase inhibitor carmofur alters fecundity in *D*. *magna*

Because sphingomyelins and sphingosine are associated with reproduction and development [62, 63] and there are changes in the expression of sphingomyelinase and ceramidase, we investigated whether the neutral sphingomyelinase inhibitor, GW4869, the acid sphingomyelinase inhibitor, zoledronic acid, or the ceramidase inhibitor, carmofur repress fecundity. Acute toxicity tests demonstrate that GW4869 caused no death at concentrations up to 1 mg/L (1.73 μM), zoledronic acid caused only 10% death at concentrations up to 1 mg/L (3.68 μM), and carmofur did not induce death until 1 mg/L (3.89 μM) with an LC50 estimated at 859 μg/L.

**Fig 10:**
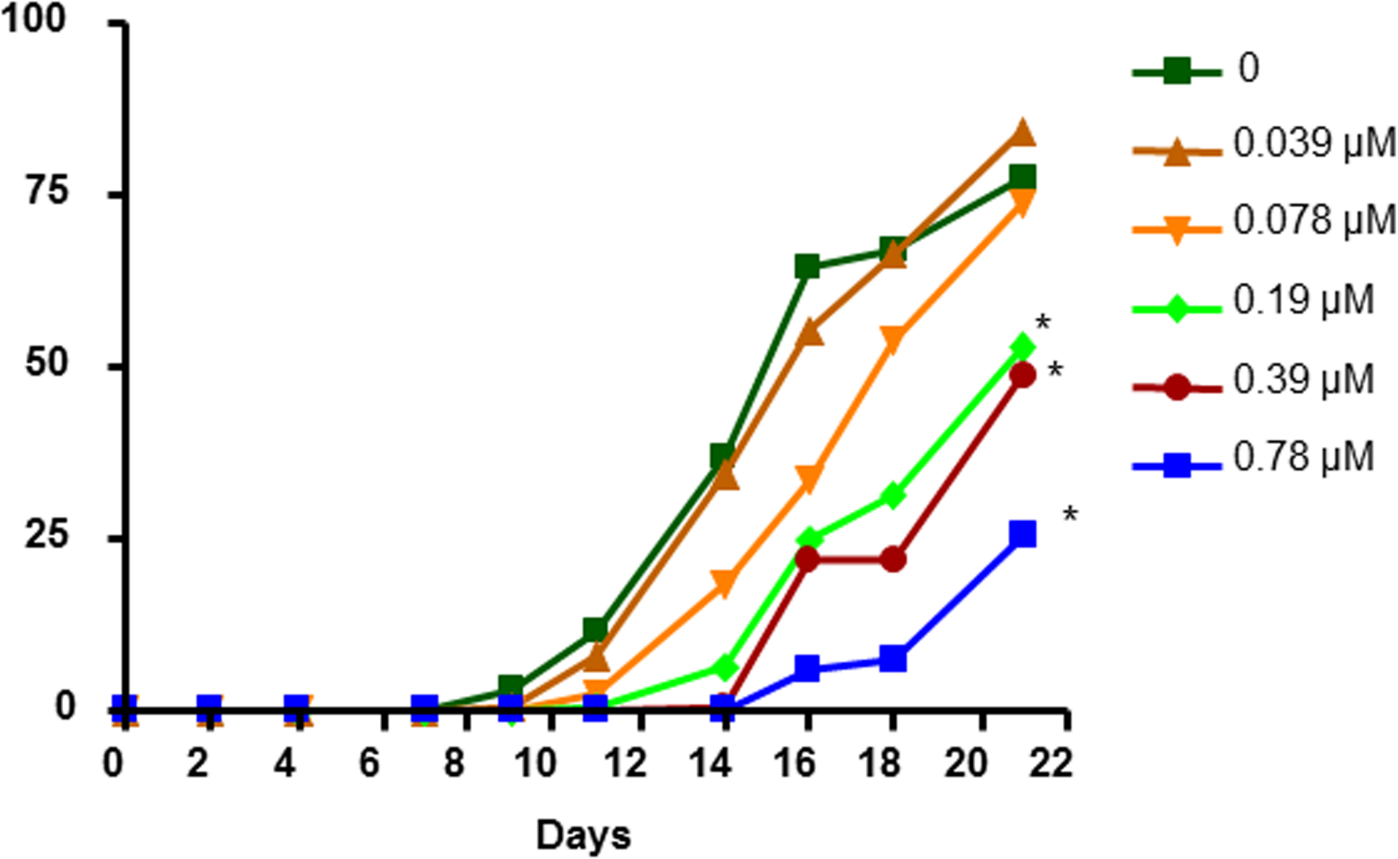
Altered fecundity of adult daphnids exposed to the ceramidase inhibitor, carmofur. Statistical significance was determined by one-way ANOVA followed by Fisher’s LSD with *p<0.001 considered significant (n = 10).

Neither the sphingomyelinase inhibitor, GW4869 nor zoledronic acid significantly perturbed reproduction (**S1 Fig**). Survival was consistent across all treatments (**S2 Fig**). However, the ceramidase inhibitor carmofur reduced reproduction in a concentration-dependent manner due to a delay in maturation and reproduction (**Fig 10**). Instead of initial reproduction occurring on day 9 as in controls, 200 μg/L (0.78 μM) carmofur-treated daphnids did not reproduce until day 16 with fecundity reduced 70%. This data suggests that reduced production of sphingosine from SMs causes delayed maturation of adults leading to lower reproduction (**Fig 7**).

## Discussion

Sphingomyelin production is age specific. The percentage of SMs in polar lipids is 8.1X higher in neonatal daphnids than adults (**Fig 3**). This is in agreement with previously published research indicating high sphingomyelin levels in neonatal daphnids relative to adults [64]. Several SMs are specific to neonates, including 36:0, 36:1, 38:1 and 38:2 (**Fig 4**). This suggests a key role for several SM species in maturation and development. We suggest that reduced metabolism of SMs into ceramides or sphingosine is associated with delayed maturation and reduced fecundity.

SMs are key polar lipids involved with cell signaling, growth and development [65]. They are converted to ceramide by sphingomyelinases, and ceramides are converted to sphingosine (involved in reproduction) by ceramidases (**Fig 7**)[47, 65]. Ceramides, SMs, sphingosine and sphingosine-1-phosphate are involved in reproduction and development in *Drosophila* [63, 66], *C*. *elegans* [65], and mice [67], and based on our data, associated with and used during development of neonatal *Daphnia*. HR96 regulates several genes in the uptake of lipids and the metabolism of SM (**Fig 7**), including magro, NPC1b, a cholesterol transporter, and two different sphingomyelinases [44, 60, 68]. From this we predict that increased sphingomyelinase activity (i.e. atrazine, age) would increase ceramide and the increased ceramidase would increase sphingosine concentrations; decreased sphingomyelinase activity would increase SMs (i.e. triclosan) and decrease ceramides [47].

We tested four HR96 regulators, of which the HR96 inhibitors DHA and triclosan significantly increased SMs. Triclosan down-regulated sphingomyelinase as expected (**Fig 8**) given that the lipidomics showed increased SM levels in neonates (**Figs 3 and 4**). It is also possible that triclosan may directly inhibit sphingomyelinase, ceramidase, or other key enzymes involved in the metabolism or production of SM leading to triclosan’s induction of SM levels in neonates. Triclosan has previously been observed to disrupt lipid metabolism through inhibition of fatty acid synthase (FASN) [69], and in turn triclosan could decrease the accumulation or distribution of lipids through this mechanism in addition to disruption of HR96’s transcriptional activity. However, previous research indicates that de novo fatty acid synthesis is low in adult *Daphnia* and thus *Daphnia* fatty acid profiles primarily reflect the contents of the diet with limited exceptions, including arachidonic acid, eicosapentaenoic acid, and probably the sphingomyelins in neonates (**Fig 3**)[12, 15, 64]. Interestingly, recent studies with *Daphnia magna* demonstrate that eggs do not contain appreciable levels of sphingomyelin [17, 70], which indicates that we cannot extrapolate our results to eggs because they don’t contain sphingomyelin and there is stage dependent de novo synthesis of sphingomyelins in *Daphnia magna*. Overall, adults treated with triclosan have the highest measured lipid levels following chemical exposure and their neonates the lowest (**Tables 1 and 2**), suggesting that in addition to reduced SM metabolism, lipids are not being allocated to the neonates in triclosan-exposed daphnids.

The physiological effects of triclosan on neonates (**Fig 2**) and adolescents [13] are only manifested under resource-limited conditions, potentially due to competition from dietary lipid sources. Delayed maturation and reproduction may have significant environmental consequences under resource-limited conditions. Considering that these conditions are not common in laboratory studies but relatively common in the environment, anthropogenic aquatic concentrations of triclosan could significantly perturb fecundity.

Given that the metabolites of SMs such as ceramides and sphingosine control several developmental processes in animals [65]; we hypothesized that the increase in SMs may be caused by reduced production of ceramide and sphingosine, and this in turn could lead to poor development. It was unknown if blocking the production of these enzymatic pathways has physiological consequences in *D*. *magna*. Interestingly, carmofur, a ceramidase inhibitor shows similar physiological effects to triclosan with delayed maturation and poor reproduction (**Fig 10**). This suggests that similarly, the increase in sphingomyelins and delayed development and reproduction [13] is a by-product of triclosan’s perturbation of sphingomyelinase and cermamidase. The inability to produce sphingosine or sphingosine-1-phosphate could be the likely culprit for the subsequent lack of development observed in neonates [13], the delayed development observed in adolescents (**Figs 2 and 10**), and the subsequent reduction in reproduction (**Figs 2 and 10**). Follow up assays to measure sphingosine-1-phosphate and sphingosine were not sensitive and further assay development and a large number of neonatal *Daphnia* are needed for future studies.

DHA, which is considered a key component of a healthy algal diet for *Daphnia* [71], also altered gene expression of key HR96-regulated genes. DHA shows a mixed type response including magro, mannosidase, and NPC1b, but not sphingomyelinase. DHA appears to regulate these early responses that regulate lipid and mannose uptake but have little effect on the specific metabolism of sphingomyelins based on the gene expression data (**Fig 8**). Further, it is interesting that that in general the lipid’s (LA and DHA) responses are usually earlier than the anthropogenic chemicals, triclosan and atrazine (**Figs 8 and 9**). DHA exposure was associated with an increase in reproduction and shorter lifespan (**Fig 2**). The mechanism is unknown, but DHA may be providing the necessary dietary lipids for the production of ceramides and sphingosine and in turn repressing the then unnecessary enzymes in SM metabolism. For example, in rats, ceramides produced by DHA down-regulate CYP2B1 through inhibition of CAR, HR96’s ortholog, and this effect is reversed by sphingomyelinase inhibitors [72]. Similar inhibition of metabolism by DHA may allow for the retention of SM for other purposes while still producing the necessary ceramides for reproduction and development.

In contrast to triclosan and DHA, atrazine induces the expression of HR96, NPC1b, sphingomyelinase and ceramidase in 11-day old *D*. *magna* (**Fig 9**), consistent with HR96 activation [41, 60]. Previous work demonstrates that atrazine provides protection from the HR96 inhibitors, DHA and triclosan, potentially through HR96 activation and increased glutathione S-transferase activity [50]. However, atrazine has no effect on magro, a key biomarker of HR96 activation [44]. This may be an example of a chemical specific effect on transcription. Another example of chemical specific effects on transcription includes bilirubin activation of CAR that does not cause induction of Cyp2b10 [73]. Overall, atrazine induced the expression of HR96 biomarker genes, including several that regulate SM metabolism.

LA induces the expression of magro and represses mannosidase and NPC1b expression; key genes in the HR96 energy utilization pathway. However, LA does not increase SM concentrations and LA-treated neonatal daphnids have the lowest SM levels. Overall, LA exposure is not associated with SM, but instead PC in neonates (**Fig 5**), a precursor to SM. LA shows HR96-like activation patterns but they are not identical to atrazine. This is similar to DHA, which shows similar but not identical gene expression changes to the anthropogenic chemical, triclosan. It is possible that LA has chemical specific effects on the HR96 receptor; however it is probably more likely that LA is activating multiple targets that may include HNF4α, another known LA target [33]. Ultimately, both LA and atrazine exposed daphnids show poor fecundity.

Low food resources such as diet restriction play a positive role in increasing lifespan of some but not all species [74, 75]. During food shortage organisms often allocate resources towards survival and somatic maintenance until normal conditions are available for development or reproduction. Previous work demonstrated that adolescent daphnids pre-exposed to triclosan and then starved did not develop into adulthood and entered a senescent-like state [13]. Here, we demonstrate that adults exposed to triclosan and then starved released neonates that are resistant to starvation possibly because of their ability to enter senescence (**Fig 2**). The mechanism for this is unknown; however, it is interesting to speculate that disruption of SM metabolism plays a role. In our current study maternal exposure to triclosan led to moderate reproduction (lower than the control group) and higher survival among neonates (**Fig 2**) despite low lipid levels (**Table 2**). Both studies show that triclosan leads to resource allocation towards survival and away from reproduction in young daphnids. The survival is clearly a benefit to the population in the short-term; however, the loss of development may severely impact the population in the future if food resources do not become available.

Opposingly, the n-6 fatty acid, LA was associated with poor reproduction. The broods from LA-exposed adults had higher lipid levels and therefore should have been able to better deal with some stressors; however this did not include neonatal starvation survival. This is somewhat surprising given the excess lipids in the LA-exposed daphnids; however previous research demonstrated that *Daphnia* species adjust their optimum offspring number depending on the food environment to maximize the fitness of the young. Thus, under ideal dietary conditions Daphnia produce many but smaller offspring and under poor dietary conditions *Daphnia* produce few but larger offspring [76, 77]. Our research indicates that LA is seen as a poor dietary resource and DHA as a more ideal dietary resource based on reproduction. However, neonate starvation survival was poor in both cases indicating that the presence of high lipid content in the diet produced offspring ill-suited for starvation survival; possibly due to a signal suggesting ideal dietary or lipid storage conditions that abruptly ended. We suspect that the dietary resources available (i.e. n-6 vs. n-3) not just the amount of dietary resources available provide the organism direction in how to respond reproductively and this signal is complex.

Toxicants can also alter energy utilization. Activating key xenobiotic sensing transcription factors such as CAR, PXR, and AhR in mammals alters energy utilization and is associated with alterations in lipid allocation sometimes resulting in obesity [34–36, 78]. Activation of HR96 in daphnids could do the same. HR96 activators such as LA and atrazine reduced reproduction, while the HR96 inhibitor, DHA increased reproduction. We propose that some of the adverse effects observed are consistent with changes in SM metabolism as both neonatal SM levels and the expression of key HR96 regulated genes are altered in chemical and age-dependent mechanisms. Of special interest was the senescent like behavior observed in triclosan pre-treated daphnids that are starved.

In conclusion, it is interesting to hypothesize that perturbations in SM metabolism caused by chemical exposures may be associated with greater longevity or poor development. In addition, fatty acids and toxicants alter energy homeostasis and in some cases probably through similar pathways. Thus, toxicants can cause sublethal effects by altering resource allocation, metabolism of signaling lipids, and therefore perturb survival, development, and reproduction under resource limited conditions. This effect, as observed with triclosan, could have under appreciated consequences on the population.

## Supporting information

S1 Table Primers used to quantify gene expression by qPCR. (PDF)

S2 Table All measured polar lipid species. (XLSX)

S1 Fig Fecundity of D. magna during 21-day chronic toxicity tests with carmofur, GW4869, and zoledronic acid. Number of neonates released per adult daphnid following exposure to (A) the ceramidase inhibitor Carmofur, (B) the neutral sphingomyelinase inhibitor GW4869, or (C) the acid sphingomyelinase inhibitor zoledronic acid. Only carmofur significantly perturbed reproduction. Statistical significance determined by one-way ANOVA followed by Fisher’s LSD used as the post-hoc test (p ≤ 0.001) (GraphPad Prism 6, GraphPad Software).
(PDF)

S2 Fig Daphnid survival during 21-day chronic toxicity tests with carmofur, GW4869, and zoledronic acid. Percent survival of daphnids exposed to (**A**) the ceramidase inhibitor Carmofur, **(B)**, the neutral sphingomyelinase inhibitor GW4869, or the **(C)** acid sphingomyelinase inhibitor zoledronic acid. Percent survival was not altered in this study. Statistical analysis was performed using Fisher’s exact test (2x2).
(PDF)
