# Supplementary material for: Exchange of polar lipids from adults to neonates in Daphnia magna: Perturbations in sphingomyelin allocation by dietary lipids and environmental toxicants
Source: PLoS One. 2017 May 24;12(5):e0178131. doi: 10.1371/journal.pone.0178131 (PMC5443554; doi:10.1371/journal.pone.0178131)
Supplement: S1 Table — (PDF) [file pone.0178131.s001.pdf]

S1 Table: **Primers used to quantify gene expression by qPCR**

Gene / ID / (Amplicon)	Primer set	Temp(°C)
HR96<br>Dapma7bEVm004188t1<br>(86 bp)	Forward 5'-GCG-GAG-ACA-AGG-CTT-TAG-GTT-3'<br>Reverse 5'-AGG-GCA-TTC-CGT-CTA-AAG-AAG-GCT-3'	58
Magro (CG5932)<br>Gastric TAG lipase<br>Dapma7bEVm018418t1<br>(108 bp)	Forward 5'-GCA-TAG-GAC-GTG-AGA-TGG-TTA-G-3'<br>Reverse 5'-ACA-AGA-AGC-TCG-CAT-GGT-TA-3'	51
lysosomal mannosidase<br>Dapma7bEVm007411t1<br>(98 bp)	Forward 5'-GGT-TCC-CTG-GAG-TTT-ATG-GTA-G-3'<br>Reverse 5'-AGT-CGT-CGG-TGA-ATC-TGT-TG-3'	53
Niemann-Pick C 1b<br>(NPC1b)(106 bp)<br>Dapma7bEVm629793t1<br>Dapma7bEVm000442t1	Forward 5'-TCA-TAG-GTG-GAC-AGC-AAG-ATT-AC-3'<br>Reverse 5'-TAG-CAG-GCA-CAC-CAA-CAT-AG-3'	55
ceramidase<br>(Cer2)(165 bp)<br>Dapma7bEVm000393t1	Forward 5'-GTG-CCT-TGT-GTA-AAG-TCG-AAA-C-3'<br>Reverse 5'-GGC-CAA-CCA-CTG-TGA-AAT-TAT-G-3'	60
Sphingomyelinase 3<br>(SM3)<br>Dapma7bEVm001825t1<br>(148 bp)	Forward 5'-GCG-CTC-TTC-CAG-CTC-TAT-TT-3'<br>Reverse 5'-GAC-GGA-TTT-GCT-CGC-ATT-TG-3'	60
β-actin<br>Dapma7bEVm018420t1<br>(165 bp)[1]	Forward 5'-CCA-CAC-TGT-CCC-CAT-TTA-TGA-AG-3'<br>Reverse 5'-CGC-GAC-CAG-CCA-AAT-CC-3'	52.2

1. Heckmann L-H, Connon R, Hutchinson TH, Maund SJ, Sibly RM, Callaghan A. Expression of target and reference genes in *Daphnia magna* exposed to ibuprofen. BMC Genomics 2006; 7: 175.
