# Supplementary material for: Exchange of polar lipids from adults to neonates in Daphnia magna: Perturbations in sphingomyelin allocation by dietary lipids and environmental toxicants
Source: PLoS One. 2017 May 24;12(5):e0178131. doi: 10.1371/journal.pone.0178131 (PMC5443554; doi:10.1371/journal.pone.0178131)

S1 Fig: Fecundity of D. magna during 21-day chronic toxicity tests with carmofur, GW4869, and zoledronic acid. Number of neonates released per adult daphnid following exposure to (**A**) the ceramidase inhibitor Carmofur, **(B)** the neutral sphingomyelinase inhibitor GW4869, or **(C)** the acid sphingomyelinase inhibitor zoledronic acid. Only carmofur significantly perturbed reproduction. Statistical significance determined by one-way ANOVA followed by Fisher's LSD used as the post-hoc test ($p \leq 0.001$) (GraphPad Prism 6, GraphPad Software).

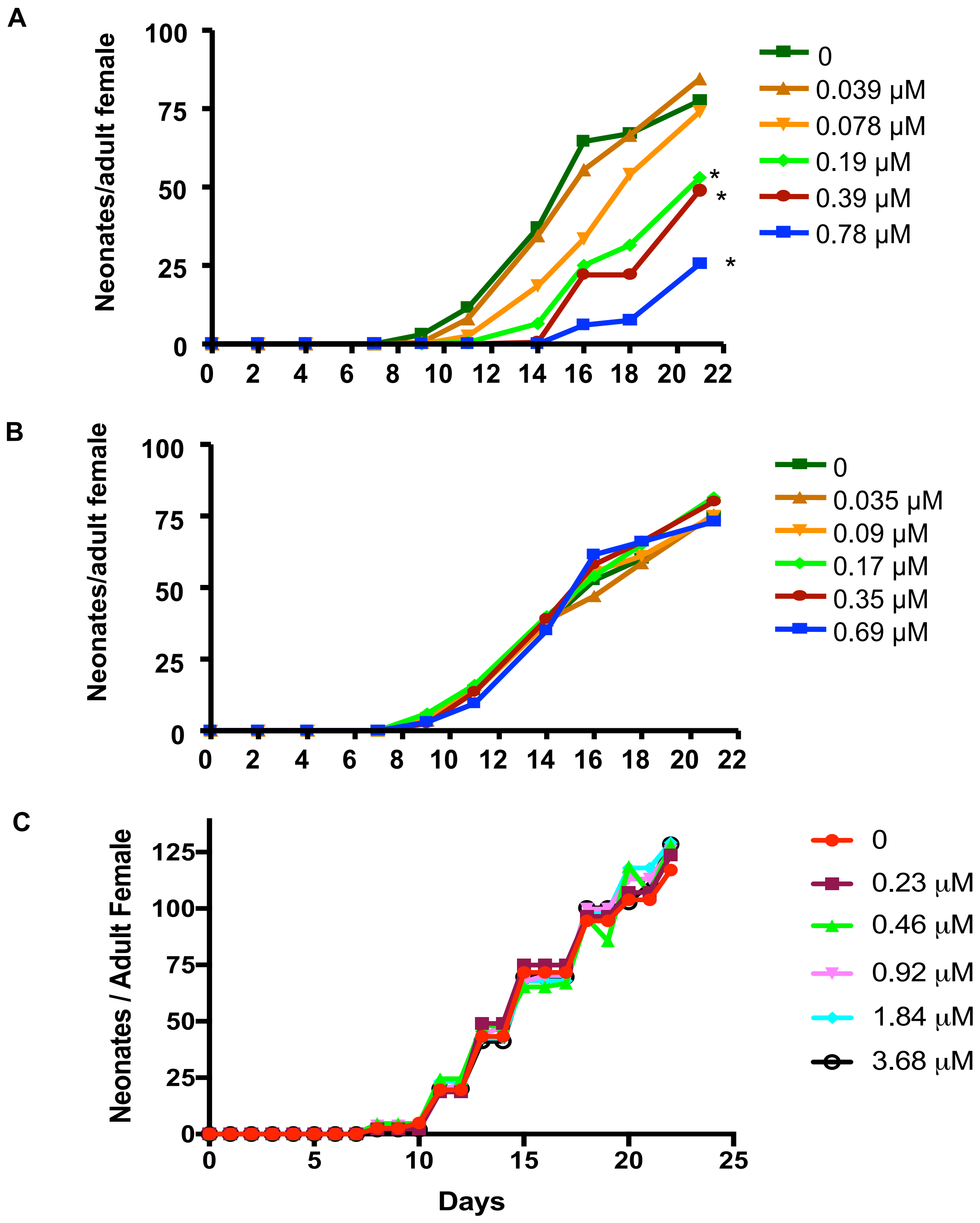

Supplement: S1 Fig — Number of neonates released per adult daphnid following exposure to (A) the ceramidase inhibitor Carmofur, (B) the neutral sphingomyelinase inhibitor GW4869, or (C) the acid sphingomyelinase inhibitor zoledronic acid. Only carmofur significantly perturbed reproduction. Statistical significance determined by one-way ANOVA followed by Fisher’s LSD used as the post-hoc test (p ≤ 0.001) (GraphPad Prism 6, GraphPad Software). (PDF) [file pone.0178131.s003.pdf]
