# Supplementary material for: Exchange of polar lipids from adults to neonates in Daphnia magna: Perturbations in sphingomyelin allocation by dietary lipids and environmental toxicants
Source: PLoS One. 2017 May 24;12(5):e0178131. doi: 10.1371/journal.pone.0178131 (PMC5443554; doi:10.1371/journal.pone.0178131)

S2 Fig: Daphnid survival during 21-day chronic toxicity tests with carmofur, GW4869, and zoledronic acid. Percent survival of daphnids exposed to (**A**) the ceramidase inhibitor Carmofur, **(B)**, the neutral sphingomyelinase inhibitor GW4869, or the **(C)** acid sphingomyelinase inhibitor zoledronic acid. Percent survival was not altered in this study. Statistical analysis was performed using Fisher's exact test (2x2).

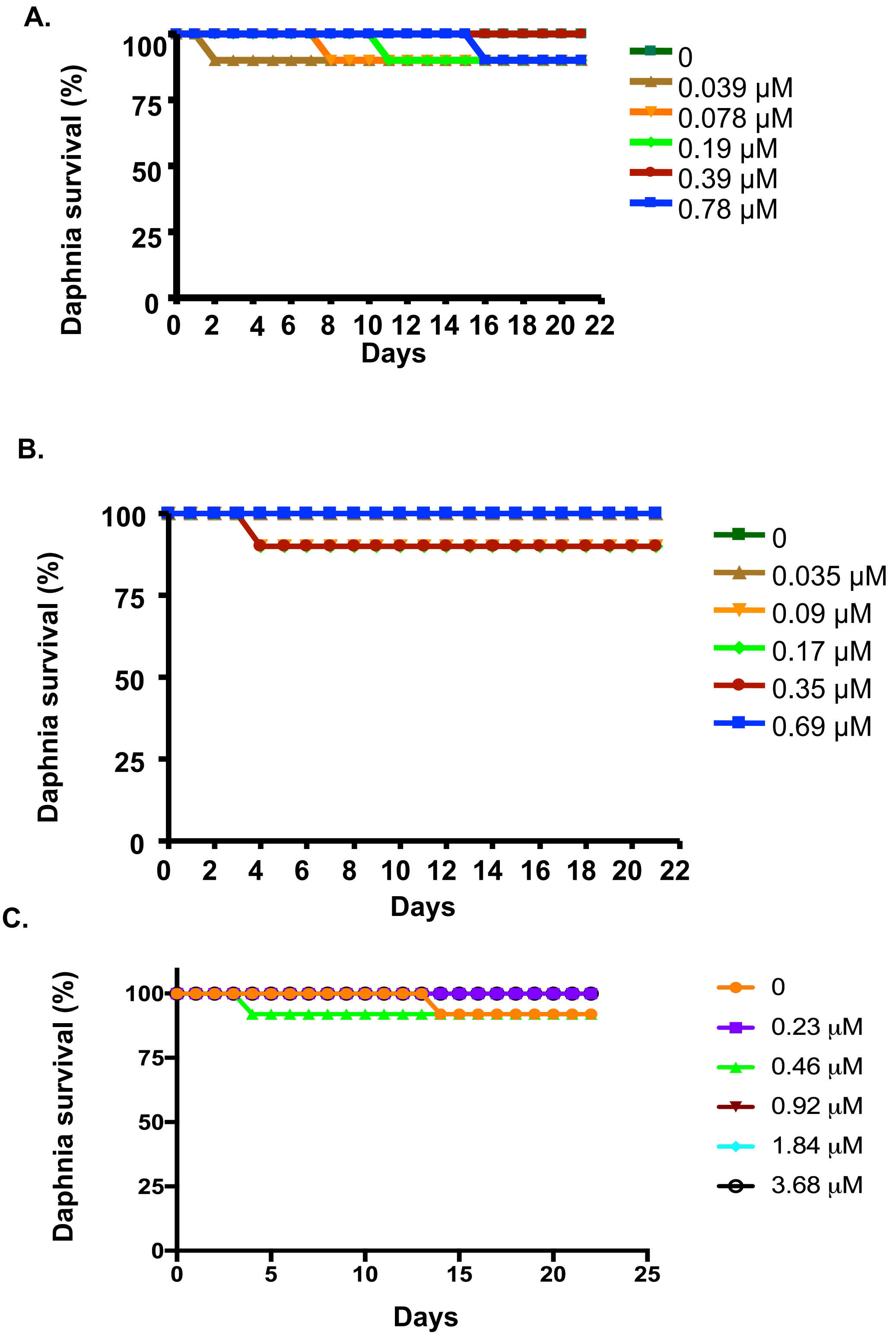

Supplement: S2 Fig — Percent survival of daphnids exposed to (A) the ceramidase inhibitor Carmofur, (B), the neutral sphingomyelinase inhibitor GW4869, or the (C) acid sphingomyelinase inhibitor zoledronic acid. Percent survival was not altered in this study. Statistical analysis was performed using Fisher’s exact test (2x2). (PDF) [file pone.0178131.s004.pdf]
